# The Relationship between Body Mass Index and Hospitalisation Rates, Days in Hospital and Costs: Findings from a Large Prospective Linked Data Study

**DOI:** 10.1371/journal.pone.0118599

**Published:** 2015-03-04

**Authors:** Rosemary J. Korda, Grace Joshy, Ellie Paige, James R. G. Butler, Louisa R. Jorm, Bette Liu, Adrian E. Bauman, Emily Banks

**Affiliations:** 1 National Centre for Epidemiology and Population Health, The Australian National University, Canberra, Australian Capital Territory, Australia; 2 Australian Centre for Economic Research on Health, The Australian National University, Canberra, Australian Capital Territory, Australia; 3 Centre for Health Research, The University of Western Sydney, Sydney, New South Wales, Australia; 4 School of Public Health and Community Medicine, The University of New South Wales, Sydney, New South Wales, Australia; 5 The Sax Institute, Sydney, New South Wales, Australia; 6 School of Public Health, The University of Sydney, Sydney, New South Wales, Australia; Erasmus University Rotterdam, NETHERLANDS

## Abstract

**Background:**

Internationally there is limited empirical evidence on the impact of overweight and obesity on health service use and costs. We estimate the burden of hospitalisation—admissions, days and costs—associated with above-normal BMI.

**Methods:**

Population-based prospective cohort study involving 224,254 adults aged ≥45y in Australia (45 and Up Study). Baseline questionnaire data (2006-2009) were linked to hospitalisation and death records (median follow-up 3.42y) and hospital cost data. The relationships between BMI and hospital admissions and days were modelled using zero-inflated negative binomial regression; generalised gamma models were used to model costs. Analyses were stratified by sex and age (45-64, 65-79, ≥80y), and adjusted for age, area of residence, education, income, smoking, alcohol-intake and private health insurance status. Population attributable fractions were also calculated.

**Results:**

There were 459,346 admissions (0.55/person-year) and 1,483,523 hospital days (1.76/person-year) during follow-up. For ages 45-64y and 65-79y, rates of admissions, days and costs increased progressively with increments of above-normal BMI. Compared to BMI 22.5-<25kg/m^2^, rates of admissions and days were 1.64-2.54 times higher for BMI 40-50kg/m^2^; costs were 1.14-1.24 times higher for BMI 27.5-<30kg/m^2^, rising to 1.77-2.15 times for BMI 40-50kg/m^2^. The BMI-hospitalisation relationship was less clear for ≥80y. We estimated that among Australians 45-79y, around 1 in every 8 admissions are attributable to overweight and obesity (2% to overweight, 11% to obesity), as are 1 in every 6 days in hospital (2%, 16%) and 1 in every 6 dollars spent on hospitalisation (3%, 14%).

**Conclusions:**

The dose-response relationship between BMI and hospital use and costs in mid-age and older Australians in the above-normal BMI range suggests even small downward shifts in BMI among these people could result in considerable reductions in their annual health care costs; whether this would result in long-term savings to the health care system is not known from this study.

## Introduction

Obesity is a major global health concern. Rates of obesity have doubled or tripled in many countries over the past three decades, and in almost half of all Organisation for Economic Co-operation and Development countries 50% or more of the population is overweight or obese [1,2]. In Australia, 63% of adults were classified as overweight (body mass index (BMI) ≥25 kg/m^2^) or obese (BMI≥30 kg/m^2^) in 2011–12 [3], and the proportion of adults who are obese has increased over time, up from 19% in 1995 to 28% in 2011–12. The proportion of adults with very high BMI (>35 kg/m^2^) doubled from 5% to 10% over the same period [3].

The effect of the obesity epidemic on health care use and expenditure is of particular concern. Primarily due to the increased risk of chronic diseases, such as diabetes, cardiovascular disease, osteoarthritis and many cancers [4–6], obesity has been linked to excess use of hospital and other health services, and consequently excess health care costs, in several countries, including Australia [7–18]. Nevertheless, there is a paucity of reliable data world-wide on the impact of overweight and obesity on health resource use and, in particular, associated costs [19]. Existing studies on costs have used varying methodologies; many have used a broad, top-down approach [19,20], allocating costs to specific diseases associated with obesity, while fewer studies have used individual-level empirical data and prospective data. Further, with some exceptions [21,22], published cost estimates are largely based on broad BMI groupings and many studies report only on obese groups; however, the overweight-but-not-obese groups are likely to contribute materially to the burden [12]. Finally, the patterns of health service use in relation to BMI vary considerably with age and sex [23,24], and the vast majority of studies have had insufficient power to adequately quantify this variation, particularly in elderly populations, nor to adjust for other factors to enable appropriate attribution of costs.

In Australia, hospitalisation accounts for the largest share of health care costs, around 40% of total recurrent health expenditure [25]. The aim of this study was to use large-scale, population-based survey data linked prospectively to hospital admission records to estimate the burden of hospitalisation—including total admissions, days in hospital and costs—associated with each narrow increment of above-normal BMI, in mid-age and older Australians.

## Materials and Methods

### Study population and data sources

We used data from the Sax Institute’s 45 and Up Study, a prospective cohort study involving 267,153 men and women aged 45 years and over from New South Wales (NSW), Australia. Participants in the 45 and Up Study were randomly sampled from the database of Australia’s universal health insurance provider, Medicare, which provides virtually complete coverage of the general population. Around 10% of the entire NSW population aged 45 years and over was included in the cohort [26]. Participants joined the study by completing a baseline questionnaire, distributed between 1 January 2006 and 31 December 2008, and provided signed consent for linkage of their information to a range of health databases. The 45 and Up Study is described in detail elsewhere [26], and questionnaires can be viewed at http://www.45andup.org.au.

For our study, baseline survey data were linked by the NSW Centre for Health Record Linkage to individual hospitalisation data from the NSW Admitted Patient Data Collection (to 31 December 2011), which contains records of all hospitalisations in NSW. These data include the Australian Refined Diagnosis Related Group (DRG) code for each admission [27]. Each DRG represents a class of patients with similar clinical conditions requiring similar hospital resources. Data were also linked to death registrations (to 31 December 2011), used in this study for censoring purposes; and cancer registrations from the NSW Central Cancer Registry (January 2000–date of baseline survey) to ascertain cancer history.

Participants were followed from the date of recruitment to either December 2011 (the last date to which hospital data were available) or date of death, whichever occurred first. Over the relatively short follow-up period, a small but unknown number of participants are likely to have moved out of NSW. Follow-up for hospitalisations is considered to be ~98% complete among those continuing to reside in NSW [28]. Quality assurance data on the data linkage show false positive and negative rates of <0.5% and <0.1%, respectively.

Participants were excluded from the analyses if they had: missing data on date of entry into the study or BMI at baseline; a single record with DRG code/cost information missing; invalid death or hospitalisation dates; or a cancer diagnosis within the five years before enrolment (excluding non-melanoma skin cancer), ascertained from the cancer registry (see S1 Fig.). Eligible participants contributed person-years from the date of recruitment until the date of death or end of follow up (31 December 2011), whichever was the earliest.

**Hospitalisation outcomes**. We investigated three outcomes: (i) total hospital admissions; (ii) total days in hospital; and (iii) total hospitalisation costs (2009–10 Australian dollars). Where patients had been transferred between hospitals, the relevant admission records were first merged together to avoid double counting of unique hospital episodes. Total days in hospital was based on length of stay (LOS, discharge date minus admission date, plus one day for same day admissions), summed for all admissions. To assign costs, we matched the DRG codes in our data to DRG-based cost data from the National Hospital Cost Data Collection Public Sector Estimated Cost Weights Reports (NHCDC) [29]. The NHCDC contains, amongst other information, average costs per DRG, based on patient-costed and cost-modelled information. We used the average DRG-specific total cost per admission in the Round 14 (2009–10) NHCDC to assign costs to each admission in our dataset (version 5.2 for admissions from January 2006 to December 2009 and version 6.0x for admissions from January 2010 to December 2011) [29]. We assigned all costs using the Public Sector reports regardless of whether the admission was a private or public hospital admission as the Private Sector reports do not include all component costs.

**Body mass index**. The main exposure, BMI, was calculated from weight and height, which were self-reported on the baseline questionnaire. Consistent with established methods [30], people with extreme measures of BMI (<15 kg/m^2^ or BMI>50 kg/m^2^) were excluded due to the increased probability of measurement error. BMI was then categorised using the following cut-points (World Health Organization (WHO) weight classification [31] in brackets): 15–18.49 (underweight), 18.5–19.99, 20–22.49 and 22.5–24.99 (normal weight), 25–27.49 and 27.5–29.99 (overweight), 30–32.49 and 32.5–34.99 (obese class I), 35–39.99 (obese class II) and 40–50 (obese class III).

### Covariates

Covariates used in modelling, obtained from the baseline questionnaire (apart from area of residence, which was obtained from Medicare records), included age, sex, area of residence (categorised as major city, inner regional, more remote, based on the Accessibility/ Remoteness Index of Australia Plus [32] score associated with the postcode of residence); education (no school qualification, school certificate/trade/ apprenticeship, certificate/diploma/degree); pre-tax annual household income (<$20,000, $20,000–29,999, $30,000–39,999, $40,000–49,999, $50,000–69,999, ≥$70,000 AUD), smoking (never, past, current), alcohol intake (0, 1-<15 and ≥15 drinks per week), and private health insurance (additional to universal health insurance, categorised as yes, no). Participants with any missing values on any of these variables were assigned to a separate category for that variable in the analysis.

### Statistical analysis

For each BMI category, we calculated total admissions, days in hospital and costs per person-year. Rates were age-standardised to the 2006 NSW population, in 5 year age-groups, using the direct method [33]. We modelled the relationship between BMI and each of the outcomes in separate models, by sex and age group (45–64, 65–79 and ≥80 years), adjusting for age at baseline (5-year age groups) and all other covariates. For the ≥80 years group, we combined the two highest BMI categories due to small numbers. For admission rates and hospital days we used zero-inflated negative binomial regression, with robust standard errors. For hospital costs we used generalised linear models assuming a gamma distribution, with the selection of the specific link functions guided by the fit of the data for each model; non-parametric bootstrapping was used to generate confidence intervals [34]. Predicted estimates based on the fitted models were calculated using the recycled predictions method, yielding adjusted average outcomes per BMI category (i.e., assumes the same distribution of covariates in all BMI categories as in the entire (age-sex specific) sample), with adjusted relative rates (RRs) estimated using 22.5-<25 kg/m^2^ as the reference category. Tests for trend can also be performed by modelling median values of the BMI categories as an ordinal variable. However, these were not performed as cumulative residual plots used to investigate the linear functional form of BMI indicated that the assumption about the linear functional form of BMI was often not satisfied. All statistical tests were two sided, using a significance level of 5%. Analyses were performed using Stata version 13.1.

Finally, we estimated the proportion of hospital admissions, days and costs associated with overweight and obesity by calculating population attributable fractions (PAFs) for those aged 45–79 years. To do this, within each age-sex group we calculated the attributable fractions among the exposed in each above-normal BMI category (using the adjusted RRs generated earlier), multiplied these by the proportion of admissions/days/costs in the corresponding BMI category (i.e. the exposure prevalence among cases (P_cases_)), (P_cases_[((RR) - 1)/RR]), then summed these for the relevant BMI categories, thus yielding internally valid PAFs for each age-sex group [35]. In addition, as the above method reflects the distribution of BMI in the study sample rather than the Australian population, instead of using the exposure prevalence among the cases, we also generated PAFs by incorporating age-sex specific BMI prevalence in the Australian population (P_pop_), using data obtained from the 2011–12 Australian Health Survey (refer to S1 Table) [36] ([P_pop_ (RR-1)/ P_pop_ (RR-1) + 1]) [35]. This method yields externally valid PAFs on the strong assumption there is no confounding or effect modification affecting the RRs. For both methods, we then weighted the age-sex-specific PAFs using the total admissions/days/costs in the Australian population in the corresponding sex-age groups in 2011–12, enabling us to estimate the overall PAFs and the absolute number of admissions, days in hospital and absolute costs associated with overweight and obesity in this combined age group (45–79 years). Data on annual admissions and days in hospital in Australia by sex and age group were obtained from published national data [28]. Data for annual hospital costs in Australia by age and sex were not available for this study. Instead, we estimated the average cost per admission by age and sex in our sample, and as these were based on 2009–10 cost data, multiplied these by a government hospital inflation factor of 3.6% [37]; we then applied these per admission costs to the total number of admissions in the Australian population in the corresponding age-sex groups to arrive at annual total costs.

Ethics approval for this project was obtained from the NSW Population and Health Services Research Ethics Committee, the University of NSW Human Research Ethics Committee and the Australian National University Human Research Ethics Committee.

## Results

After exclusions (S1 Fig.), we followed 224,254 participants (84%) over 842,051 person-years, (median 3.42 years; range: 2 days to 6 years). In the final sample, 62% of participants were overweight or obese (40% and 22%, respectively), including two-thirds of men (47% overweight and 22% obese) and just over half of the women (33% overweight and 23% obese). This proportion was considerably lower in those aged 80 years or older (47% overweight/obese). Just under half of participants (47%) were men, with the majority (62%) aged 45–64 years and 10% aged 80 years or older. Further characteristics of the sample, including missing data, are shown in Table 1.

**Table 1:** Demographic and health characteristics of the sample.

	BMI category (kg/m^2^)										Total sample
	Underweight	Normal weight			Overweight		Obese				
	15-<18.5	18.5-<20	20-<22.5	22.5-<25	25-<27.5	27.5-<30	30-<32.5	32.5-<35	35-<40	40–50	
**N**	2826	6257	27 557	48 988	50 688	37 950	23 206	12 496	10 245	4041	224 254
**% of total**	1.3	2.8	12.3	21.9	22.6	16.9	10.4	5.6	4.6	1.8	100
**Male**	24.7	22.0	32.0	45.5	55.2	57.1	51.4	46.1	37.6	29.4	47.1
**Age group**											
**45–64 years**	49.6	60.3	62.1	59.7	60.1	62.1	65.1	66.9	71.6	77.4	62.2
**65–79 years**	26.3	22.6	24.4	28.2	30.2	30.5	28.7	28.2	24.8	20.1	28.1
**≥80 years**	24.1	17.1	13.5	12.1	9.7	7.5	6.3	4.9	3.6	2.6	9.7
**Lives in major city**	47.4	50.7	48.9	47.8	45.3	43.5	42.4	40.4	39.4	40.0	45.2
**Income ≥$70 000**	12.6	21.3	23.8	25.5	26.3	25.6	23.5	22.1	20.5	18.6	24.4
**University degree**	40.4	48.7	49.8	48.6	45.6	42.5	40.0	37.3	37.5	37.2	44.7
**Health insurance (yes)**	56.4	62.9	66.6	68.1	68.2	67	64.7	61.5	58.7	52.7	66.0
**Current smoker**	16.1	10.8	9.0	7.1	6.3	6.2	6.7	7.0	7.2	7.7	7.2
**Alcohol ≥15 drinks p/w**	8.5	8.2	10.3	13.4	16.4	17.3	16.3	15.0	11.9	7.9	14.4

Notes. 1.Cell numbers are percentage of the sample within each BMI category, except for % total where % refers to % of total sample in each BMI category.

2. Participants with missing values are not included in the percentages (% missing: education = 1.44%; region = 0.02%; income = 20.88% (includes refusal to disclose); smoking status = 0.30%; alcohol consumption = 1.92%; physical activity = 1.87%; other variables = <0.01%).

A summary of hospitalisations occurring following study entry, presented separately by age group and sex, are shown in S2 Table. The majority of participants (127,908, 57%) had at least one hospital admission during the follow-up period. There were a total of 459,346 admissions (0.55 per person-year), 1,483,523 days spent in hospital (1.76 per person-year) and $2,153 million in hospitalisation costs ($2,557 per person-year). Just over one third (36.5%) of admissions involved at least one overnight stay. The mean LOS per admission was 3.23 days (SD: 8.70), and mean cost per admission was $4,681.

**Fig 1:**
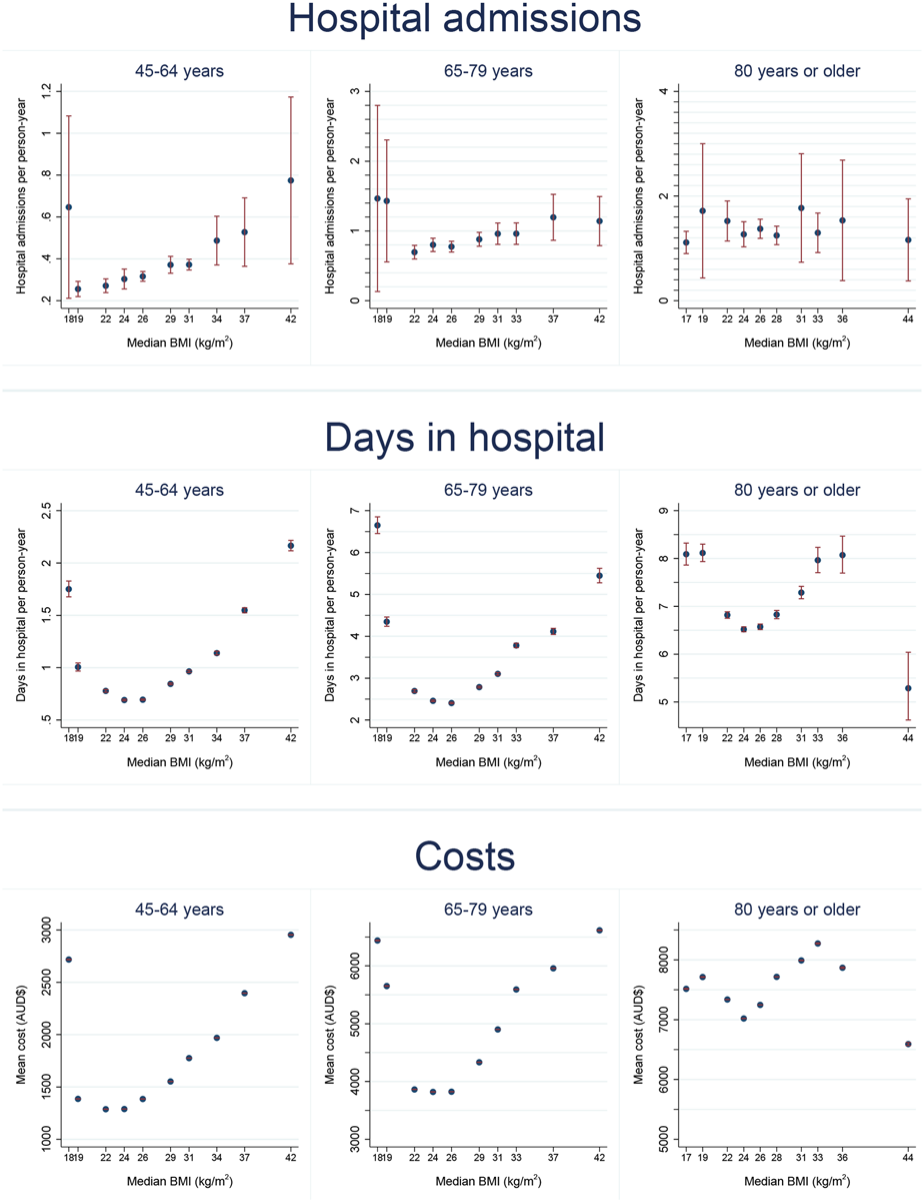
Age-standardised hospital admissions, days in hospital and costs per person-year (with 95% CIs) by body mass index (BMI), males. Notes. 1. Within each age group, rates are age-standardised to the 2006 NSW population (in 5 year age-groups) using the direct method.

**Fig 2:**
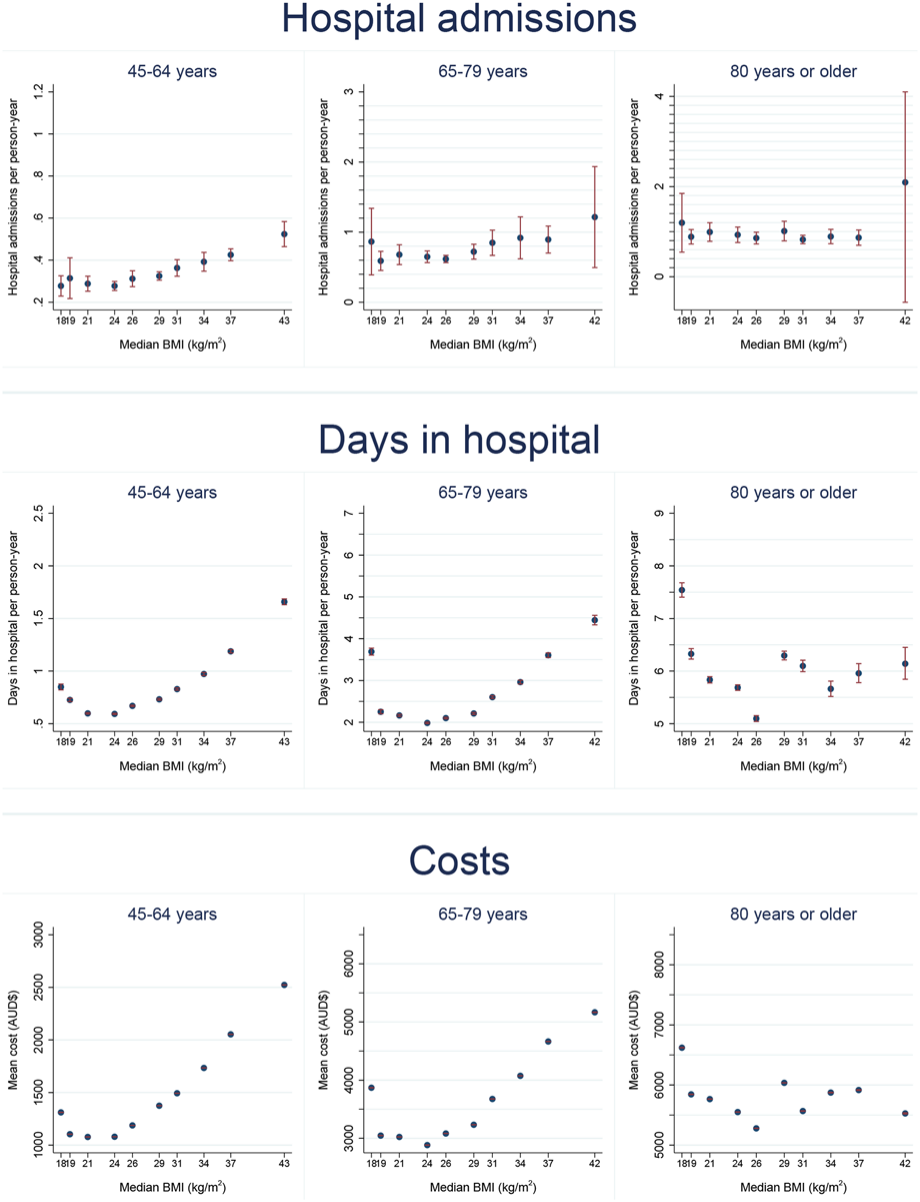
Age-standardised hospital admissions, days in hospital and costs per person-year (with 95% CIs) by body mass index (BMI), females. Notes. 1. Within each age group, rates are age-standardised to the 2006 NSW population (in 5 year age-groups) using the direct method. 2. The upper confidence interval for the 80 years or older age group for hospital admissions has been truncated to 4.1 (actual value is 4.8).

Age-standardised admissions, days in hospital and costs per person-year in relation to BMI are shown in Fig. 1 (males) and Fig. 2 (females). For men and women aged 45–64 and 65–79, the outcomes generally showed a J-shaped relationship with BMI. Rates were elevated at low BMI levels (15-<18.5 kg/m^2^, and often also 18.5-<20 kg/m^2^), and they rose steadily with increasing above-normal BMI. These same relationships were not evident in those aged 80 years or older.

### Modelled estimates

***Hospital admission rates*** in relation to BMI and associated relative rates, adjusted for all covariates, are presented in Table 2. Hospitalisation rates increased with increasing above-normal BMI (>25 kg/m^2^ in those aged 45–64 years and >27.5 kg/m^2^ in those aged 65–79 years), such that rates for those with BMI 40–50 kg/m^2^ were 1.7 to 2.2 times higher than in those with BMI 22.5-<25 kg/m^2^. Amongst those 80 years or older, patterns were less clear and estimates were not significant.

**Table 2:** Estimated hospital admission rates by body mass index (BMI)^(1)^.

	*45–64 years*		*65–79 years*		*≥80 years*	
BMI category	Average admissions per py	Relative Rate (95% CI)	Average admissions per py	Relative Rate (95% CI)	Average admissions per py	Relative Rate (95% CI)
*Males*						
15-<18.5	0.81	2.50 (0.91–6.82)	1.59	1.94 (0.85–4.43)	1.36	1.03 (0.84–1.28)
18.5-<20	0.26	0.78 (0.65–0.93)	1.47	1.79 (1.07–2.99)	1.55	1.17 (0.73–1.86)
20-<22.5	0.31	0.95 (0.81–1.12)	0.78	0.95 (0.78–1.16)	1.77	1.34 (1.04–1.71)
22.5-<25	0.33	1.00	0.83	1.00	1.33	1.00
25-<27.5	0.36	1.10 (0.98–1.24)	0.81	0.97 (0.85–1.11)	1.52	1.14 (0.94–1.38)
27.5-<30	0.42	1.24 (1.09–1.41)	0.93	1.12 (0.96–1.31)	1.40	1.05 (0.87–1.27)
30-<32.5	0.41	1.23 (1.09–1.39)	1.01	1.20 (1.02–1.42)	1.85	1.39 (0.95–2.02)
32.5-<35	0.50	1.49 (1.20–1.85)	1.03	1.23 (0.99–1.52)	1.83	1.37 (0.80–2.34)
35-<40^(2)^	0.51	1.49 (1.18–1.89)	1.30	1.57 (1.17–2.12)	2.00	1.50 (0.64–3.53)
40–50	0.73	2.19 (1.42–3.38)	1.42	1.70 (1.12–2.57)		
*Females*						
15-<18.5	0.30	0.98 (0.82–1.17)	0.89	1.31 (0.84–2.04)	1.40	1.40 (0.88–2.25)
18.5-<20	0.32	1.06 (0.82–1.38)	0.60	0.88 (0.72–1.07)	1.04	1.04 (0.77–1.41)
20-<22.5	0.30	1.01 (0.89–1.14)	0.71	1.04 (0.84–1.28)	1.09	1.09 (0.86–1.38)
22.5-<25	0.30	1.00	0.68	1.00	1.00	1.00
25-<27.5	0.33	1.10 (0.96–1.26)	0.64	0.93 (0.80–1.09)	0.93	0.93 (0.76–1.14)
27.5-<30	0.35	1.14 (1.02–1.26)	0.72	1.04 (0.86–1.26)	1.13	1.13 (0.83–1.53)
30-<32.5	0.38	1.25 (1.09–1.44)	0.85	1.23 (0.97–1.55)	0.88	0.88 (0.74–1.06)
32.5-<35	0.40	1.31 (1.16–1.47)	0.83	1.19 (0.96–1.47)	0.94	0.95 (0.76–1.20)
35-<40^(2)^	0.44	1.42 (1.27–1.58)	0.91	1.30 (1.03–1.64)	1.37	1.38 (0.70–2.70)
40–50	0.51	1.64 (1.43–1.87)	1.18	1.74 (1.00–3.01)		

py = person year

1. Within each age-sex group, adjusted for age, income, education, health insurance, region, smoking status and alcohol consumption.

2. In ≥80 years age group, this category also includes those with BMI 40–50 kg/m^2^.

***Total days in hospital per person year*** in relation to BMI, adjusted for all covariates, are presented in Table 3. The total number of days in hospital per person-year was also significantly higher in those who were underweight compared to those with BMI 22.5-<25 kg/m^2^ among males aged 45–64 and 65–79 years; also, the number of days increased incrementally with increasing above-normal BMI among both males and females in these age-groups. In those aged 80 years or older, total days in hospital per person-year were only significantly higher in the low weight groups (<20 kg/m^2^) compared to BMI 22.5-<25 kg/m^2^.

**Table 3:** Estimated days in hospital per person year, by body mass index (BMI) ^(1)^.

	*45–64 years*		*65–79 years*		*≥80 years*	
BMI category	Average days per py	Relative Rate (95% CI)	Average days per py	Relative Rate (95% CI)	Average days per py	Relative Rate (95% CI)
*Males*						
15-<18.5	1.90	2.19 (1.22–3.96)	8.97	3.02 (1.94–4.69)	14.42	1.85 (1.37–2.51)
18.5-<20	0.99	1.17 (0.82–1.65)	6.03	2.00 (1.45–2.77)	10.53	1.35 (1.06–1.71)
20-<22.5	0.95	1.17 (0.97–1.43)	3.45	1.16 (0.99–1.36)	9.25	1.17 (1.03–1.34)
22.5-<25	0.84	1.00	3.01	1.00	7.88	1.00
25-<27.5	0.92	1.08 (0.97–1.21)	2.81	0.93 (0.84–1.03)	8.01	1.01 (0.91–1.13)
27.5-<30	1.07	1.25 (1.12–1.39)	3.24	1.07 (0.96–1.19)	8.45	1.07 (0.94–1.22)
30-<32.5	1.20	1.39 (1.23–1.57)	3.65	1.19 (1.05–1.35)	9.56	1.21 (1.01–1.44)
32.5-<35	1.27	1.46 (1.25–1.70)	4.18	1.36 (1.17–1.58)	10.19	1.28 (0.97–1.70)
35-<40^(2)^	1.60	1.80 (1.49–2.18)	4.75	1.57 (1.31–1.87)	10.21	1.29 (0.93–1.78)
40–50	2.22	2.54 (1.99–3.24)	6.37	2.06 (1.58–2.69)		
*Females*						
15-<18.5	0.90	1.26 (0.96–1.65)	3.67	1.66 (1.25–2.20)	10.39	1.56 (1.27–1.92)
18.5-<20	0.80	1.13 (0.93–1.39)	2.53	1.17 (0.94–1.45)	7.57	1.14 (0.97–1.34)
20-<22.5	0.71	1.00 (0.87–1.14)	2.59	1.18 (1.03–1.36)	6.95	1.06 (0.92–1.21)
22.5-<25	0.72	1.00	2.20	1.00	6.63	1.00
25-<27.5	0.81	1.12 (0.98–1.27)	2.28	1.03 (0.90–1.18)	6.24	0.95 (0.83–1.09)
27.5-<30	0.87	1.18 (1.05–1.33)	2.35	1.05 (0.92–1.20)	7.19	1.09 (0.92–1.28)
30-<32.5	0.96	1.29 (1.13–1.48)	2.87	1.27 (1.10–1.48)	7.05	1.07 (0.89–1.30)
32.5-<35	1.08	1.45 (1.26–1.67)	3.06	1.35 (1.14–1.60)	6.47	0.98 (0.76–1.26)
35-<40^(2)^	1.26	1.69 (1.48–1.93)	3.78	1.68 (1.40–2.00)	7.45	1.13 (0.86–1.48)
40–50	1.65	2.15 (1.79–2.58)	4.99	2.24 (1.68–2.99)		

py = person year

1. Within each age-sex group, adjusted for age, income, education, health insurance, region, smoking status and alcohol consumption.

2. In ≥80 years age group, this category also includes those with BMI 40–50 kg/m^2^.

***Costs per person year*** in relation to BMI, adjusted for all covariates, are presented in Table 4. The estimates of relative costs in the overweight and obese ranges were similar for males and females in 45–64 and 65–79 age groups, being slightly elevated in the overweight range (14–24% higher for BMI 27.5-<30 kg/m^2^), around 30% higher in the class I obesity range (32.5–34.99 kg/m^2^) and rising to 80% to 115% higher amongst those with class III obesity (40–50 kg/m^2^).

**Table 4:** Estimated hospital costs per person year, by body mass index (BMI)^(1)^.

	*45–64 years*		*65–79 years*		*≥80 years*	
BMI category	Costs per py	Relative Rate (95% CI)	Costs per py	Relative Rate (95% CI)	Costs per py	Relative Rate (95% CI)
*Males*						
15-<18.5	2346	1.62 (1.08–2.32)	5939	1.59 (1.19–2.07)	7078	1.07 (0.90–1.27)
18.5-<20	1331	0.92 (0.76–1.09)	5649	1.51 (1.23–1.81)	7189	1.09 (0.96–1.24)
20-<22.5	1344	0.93 (0.86–1.01)	3769	1.01 (0.94–1.07)	7076	1.07 (1.00–1.14)
22.5-<25	1444	1.00	3745	1.00	6599	1.00
25-<27.5	1564	1.08 (1.04–1.13)	3742	1.00 (0.97–1.03)	7015	1.06 (1.01–1.12)
27.5-<30	1739	1.20 (1.15–1.26)	4289	1.15 (1.10–1.19)	7289	1.10 (1.04–1.18)
30-<32.5	1862	1.29 (1.23–1.36)	4763	1.27 (1.20–1.35)	7965	1.21 (1.09–1.34)
32.5-<35	2031	1.41 (1.30–1.53)	5369	1.43 (1.32–1.55)	7653	1.16 (1.01–1.32)
35-<40^(2)^	2401	1.66 (1.52–1.83)	5678	1.52 (1.37–1.66)	8251	1.25 (1.00–1.55)
40–50	2823	1.95 (1.65–2.28)	6644	1.77 (1.45–2.08)		
*Females*						
15-<18.5	1220	1.06 (0.89–1.24)	3614	1.31 (1.11–1.53)	6526	1.20 (1.06–1.35)
18.5-<20	1101	0.96 (0.86–1.06)	2870	1.04 (0.91–1.18)	5821	1.07 (0.97–1.18)
20-<22.5	1118	0.97 (0.92–1.02)	2946	1.07 (1.00–1.14)	5649	1.04 (0.97–1.11)
22.5-<25	1152	1.00	2764	1.00	5422	1.00
25-<27.5	1250	1.09 (1.03–1.14)	2979	1.08 (1.02–1.13)	5249	0.97 (0.91–1.03)
27.5-<30	1424	1.24 (1.18–1.29)	3144	1.14 (1.08–1.20)	5727	1.06 (0.97–1.14)
30-<32.5	1528	1.33 (1.26–1.41)	3568	1.29 (1.22–1.39)	5568	1.03 (0.92–1.13)
32.5-<35	1754	1.52 (1.42–1.63)	3766	1.36 (1.26–1.48)	5544	1.02 (0.87–1.18)
35-<40^(2)^	2015	1.75 (1.65–1.86)	4497	1.63 (1.49–1.78)	6276	1.16 (0.98–1.37)
40–50	2480	2.15 (1.98–2.35)	5032	1.82 (1.54–2.14)		

py = person year

1. Within each age-sex group adjusted for current age, income, education, health insurance, region, smoking status and alcohol consumption.

2. In ≥80 years age group, this category also includes those with BMI 40–50 kg/m^2^.

Notably, while the pattern and magnitude of RRs for admissions, days and costs in relation to BMI were similar across age and sex, the corresponding absolute differences for overweight and obese patients compared to normal weight patients were for the most part higher in men than women and substantially higher in the 65–79 age group than the 45–64 age group.

**Hospitalisation burden attributable to above-normal BMI**. Using data from the study cohort, we estimated that the following were attributable to overweight and obesity for the 45–79 age group: 11% of hospital admissions (3% to overweight and 8% to obesity), 14% of days in hospital (3% and 11%) and nearly 14% of hospital costs (around 4% and 9%) (Table 5). The prevalence of high BMI in the general Australian population in 2011–12 was somewhat higher than that in the study cohort [36]. Hence, the estimates of admissions, days and costs attributable to overweight and obesity for the general population were slightly higher: over 600,000 hospital admissions, or 1 in every 8 admissions in this age group (around 2% to overweight and 11% to obesity); over 2 million days in hospital, or one in every 6 days (2% and 16%) and around $3.8 billion in total costs, or 1 in every 6 dollars spent (3% and 14%) (Table 5).

**Table 5:** Hospitalisation burden attributable to overweight and obesity in the study cohort and the Australian population aged 45–79 years in 2011–12.

	Admissions		Days		Costs ($million)	
	PAF (%)	Total	PAF (%)	Total	PAF (%)	Total
**PAFs from study cohort**						
**Overweight**	3.02	150 819	3.04	412 769	4.10	920
**Obese**	7.63	380 660	11.22	1 522 296	9.44	2117
**Total overweight and obese**	10.66	531 478	14.26	1 935 065	13.54	3037
**PAFs incorporating 2011-12 Australian BMI prevalence data**						
**Overweight**	2.13	106 128	1.90	257 306	3.13	703
**Obese**	11.08	552 605	15.72	2 133 721	13.97	3134
**Total overweight and obese**	13.21	658 732	17.62	2 391 027	17.10	3837

## Discussion

In mid-age and older Australian adults, admission rates and total days in hospital rise with increasing above-normal BMI. These elevated rates, combined with the high prevalence of overweight and obesity in the Australian population, result in substantial excess hospital costs, estimated to be around 17% of total hospital costs, or nearly four billion dollars, in the 45–79 year age group alone in 2011–12. Although the excess burden is mostly attributed to obesity rather than overweight, the total attributed to people in the overweight range (25-<30 kg/m^2^) is still substantial, underlying the importance of including estimates for burden associated with overweight, not just obesity.

While previous studies have mostly shown increased hospitalisation rates and costs associated with obesity, their relationship with lower levels of BMI are less well established, with mixed findings regarding the excess hospitalisation rates and costs in the overweight range and in the elderly [7,17,18,24,38–40]. Some of this inconsistency may be explained by the fact that broad BMI categories are used in most studies, often necessary due to lack of power to investigate finer BMI groupings. In our large study, using finer categories of BMI, we were able to show clear patterns of increasing hospitalisation rates, days in hospital and costs with increasing above-normal normal BMI, predominantly in those aged 45–79.

Admission rates, days in hospital and hospital costs were lowest in those with a BMI range of around 20-<25 kg/m^2^. This is similar to the findings from prospective studies in other countries that examined admissions [40] and costs [21,22] in relation to narrow BMI categories. Importantly, we found that hospitalisation rates and costs in men were for the most part elevated in the low-normal BMI range (18.5-<20 kg/m^2^), relative to BMI 20-<22.5 kg/m^2^, which means if the broad WHO category of normal weight is used as the reference group (i.e. 18.5-<25 kg/m^2^) the excess risk associated with overweight and obesity may be underestimated, as has been illustrated in studies of BMI and mortality [41]. That mean costs were high in the underweight groups, particularly amongst men, is likely to reflect reverse causality (with poor health leading to weight loss). In a study comparable to ours, but where follow-up time was longer and those who died in the first five years were excluded, there were less marked (and often not significant) differences in the costs for underweight and normal weight participants [21]. That the relationship between BMI and hospitalisation use and costs is less clear in the elderly (≥80 years) also needs to be taken into account when estimating the burden attributable to high BMI in the population.

The total proportion of hospital admissions attributed to overweight and obesity in our study was substantial, at around 13% of total admissions (2% for overweight, 11% for obesity) or 1 in every 8 admissions. A recent prospective study of hospitalisation rates in the UK estimated very similar attributable fractions, 3% for overweight and 9% for obesity in the population aged 50–85 [40]. There are no directly comparable previous estimates for Australia.

While our study only examined hospitalisation rates and costs, it is likely that use of other health care resources, including ambulatory medical care and pharmaceuticals, also increase with increasing BMI. For example, an earlier study of mid-age Australian women using linked Medicare records (essentially out-of-hospital medical and pathology services), found excess costs in obese women and in sedentary overweight women [15]. International studies also showed increased costs across different types of care. In a recent empirical study of working-age adults in the US, medical (including hospital inpatient and ambulatory care) and pharmaceutical costs both rose gradually with increasing BMI, starting from a BMI of 19 kg/m^2^ [22]. Similarly, in a Japanese study, physician visits and outpatient costs rose incrementally from a BMI of 18.5–20.9 kg/m^2^, while inpatient days in hospital and costs showed a J-shaped relationship with BMI as in our study, with estimates lowest for BMI 23–24.9 kg/m^2^ [17]. In addition, there are also likely to be increased costs outside the health care sector associated with overweight and obesity, such as those related to absenteeism and lost productivity. In a previous Australian study based on retrospective self-report of service use in people aged ≥25 years, there were considerable excess costs associated with overweight and obesity for health care (hospitalisation, ambulatory services and pharmaceuticals), non-health care (e.g. transport to hospital, special food) and government subsidies (e.g. disability pension) [12].

Although difficult to compare directly across populations, health systems and time periods, our finding of 17% of hospital costs attributable to overweight and obesity is generally higher than found in studies examining total healthcare expenditures, which show attributable fractions mostly in the range of 2–4%, but as high as 12% [14,16,19,40]. Findings from international studies on total health care costs also differ in terms of the relative contributions of overweight versus obesity [16,18,40,42], with costs attributable to overweight vs obesity ranging from 3% vs 97% in a Swedish study [18] to 34% vs 66% in a Canadian study [16], compared to 20% vs 80% in our study on hospital costs.

Major strengths of our study are: (i) its large sample size, enabling examination of outcomes separately by age group and sex in relation to narrow BMI increments; (ii) the availability of data on a range of potential confounders, allowing covariate adjustments; (iii) virtually complete follow-up data on hospitalisations (and deaths for censoring); and (iv) availability of DRG-specific nationally-collected costs. There are several limitations that should be borne in mind when interpreting the results: (i) Data on exposures were mostly based on self-report; this included BMI, which was calculated using self-reported weight and height at baseline. Although people tend to underestimate their weight and overestimate their height [43], and consequently underestimate BMI, a validation study involving participants in the 45 and Up Study revealed that the mean difference between self-reported and measured BMI was not large (on average-0.74 kg/m^2^) and correlations between self-reported and measured height and weight were 0.95 and 0.99, respectively [44]; however, it is also likely that, at least in people <65 years of age, BMI at time of admission would be higher than at baseline as people in this age group tend to put on weight over time; (ii) Because a dose—response relationship in outcomes with above-normal BMI was evident after adjustment for a range of important confounders, we assumed these incremental outcomes could be attributed to above-normal BMI. This assumes full adjustment of relevant factors has been achieved, nevertheless a contribution of residual confounding to the estimates cannot be ruled out. Further, due to the relatively short follow up period, we cannot rule out the effect of pre-existing disease at baseline on BMI, although we did exclude patients with a history of cancer at baseline; (iii) The costs assigned to each individual are indicative only as they are based on the DRG-specific average costs of public hospital care and they are not sensitive to individual variations in resource use within DRG categories; (iv) While the 45 and Up cohort are broadly representative of the Australian population in this age group, they are likely to be healthier, and have lower hospitalisation rates than the general population in this age group. However, given the near-complete follow up of the cohort and other methodological considerations, internal comparisons of rates and costs are valid and care was taken to use representative population estimates of BMI when estimating population attributable fractions [45]. Consequently, we suggest more emphasis be placed on the relative, than on the absolute, rates and costs of hospital admissions; (v) Finally, these data do not allow estimation of the hospitalisation burden attributable to obesity and overweight in younger adults and children, but excess burden is likely in these age groups.

## Conclusion

Our empirical, prospective data show considerable excess annual hospital admissions, days and costs associated with above-normal BMI in mid-age and older Australians, starting in the overweight range, although uncertainties remain regarding optimal BMI in the elderly. While the total excess burden associated with overweight is substantial, the markedly elevated hospitalisation rates and costs in obese BMI groups, including class I obesity, is of particular concern. This is not only an issue for individuals in this BMI range; it places a large burden on the health system, which is likely to increase as the proportion in these higher BMI ranges has been rising [3]. The dose-response relationship between BMI and hospital costs in mid-age and older Australians in the above-normal BMI range suggests that even small downward shifts in BMI among these people could result in considerable reductions in their annual health care costs. However, whether this would result in long-term savings to the health care system is not known from this study as it was beyond the scope of this paper to model lifetime health care costs. Results from previous studies on lifetime medical costs of obesity are mixed (e.g. [46–48]).

## Supporting Information

S1 Table The proportion (%) of the Australian population in overweight and obese BMI categories in 2011–2012, separately by sex and age group. Note. Data sourced from the 2011–12 Australian Health Survey.[36]
(DOCX)

S2 Table Summary of crude data, by body mass index (BMI), separately by sex and age group. Abbreviations: BMI = body mass index; n = number of participants; py = person year. Notes. 1. Costs are derived from DRG-specific cost estimates in the 2009–10 National Hospital Cost Data Collection Public Sector Estimated Cost Weights Report (NHCDC).[29]
(DOCX)

S1 Fig Flow diagram of study exclusions. (TIF)
