# Supplementary material for: The Relationship between Body Mass Index and Hospitalisation Rates, Days in Hospital and Costs: Findings from a Large Prospective Linked Data Study
Source: PLoS One. 2015 Mar 4;10(3):e0118599. doi: 10.1371/journal.pone.0118599 (PMC4349828; doi:10.1371/journal.pone.0118599)
Supplement: S1 Table — Note. Data sourced from the 2011–12 Australian Health Survey.[36] (DOCX) [file pone.0118599.s001.docx]

**Table S1. The proportion (%) of the Australian population in overweight and obese BMI categories in 2011-2012, separately by sex and age group**

|  |  | **25-<27.5** | **27.5-<30** | **30-<32.5** | **32.5-<35** | **35-<40** | **40-50** |
| --- | --- | --- | --- | --- | --- | --- | --- |
| **Male** | **45-64 years** | 26.43 | 17.98 | 19.67 | 8.43 | 8.88 | 3.90 |
|  | **65-79 years** | 27.53 | 18.96 | 21.24 | 5.26 | 6.89 | 2.52 |
|  |  |  |  |  |  |  |  |
| **Female** | **45-64 years** | 23.68 | 12.46 | 14.87 | 7.12 | 9.47 | 4.70 |
|  | **65-79 years** | 18.88 | 17.60 | 13.69 | 7.82 | 11.84 | 4.88 |

Note. Data sourced from the 2011-12 Australian Health Survey.[[36](#_ENREF_36)]
