# Supplementary material for: The Relationship between Body Mass Index and Hospitalisation Rates, Days in Hospital and Costs: Findings from a Large Prospective Linked Data Study
Source: PLoS One. 2015 Mar 4;10(3):e0118599. doi: 10.1371/journal.pone.0118599 (PMC4349828; doi:10.1371/journal.pone.0118599)
Supplement: S2 Table — Abbreviations: BMI = body mass index; n = number of participants; py = person year. Notes. 1. Costs are derived from DRG-specific cost estimates in the 2009–10 National Hospital Cost Data Collection Public Sector Estimated Cost Weights Report (NHCDC).[29] (DOCX) [file pone.0118599.s002.docx]

**Table S2. Summary of crude data, by body mass index (BMI), separately by sex and age group**

| **MALES** |  |  |  |  |  |  |  |  |  |  |  |  |  |  |  |
| --- | --- | --- | --- | --- | --- | --- | --- | --- | --- | --- | --- | --- | --- | --- | --- |
| **BMI range**  **(kg/m^2^)** |  | **Median**  **BMI** |  | **n (%)** |  | **PYs** |  | **Admissions** | |  | **Days**  **in hospital** | |  | **Costs** | |
|  |  |  |  |  |  |  |  | **Total** | **Per py** |  | **Total** | **Per py** |  | **Total ($mil)** | **Per py ($)** |
| **45-64 years** |  |  |  |  |  |  |  |  |  |  |  |  |  |  |  |
| 15-<18.5 |  | 18 |  | 309 (1) |  | 1169 |  | 870 | 0.74 |  | 2213 | 1.89 |  | 3.42 | 2926 |
| 18.5-<20 |  | 19 |  | 661 (1) |  | 2567 |  | 692 | 0.27 |  | 2714 | 1.06 |  | 3.76 | 1465 |
| 20-<22.5 |  | 22 |  | 4330 (7) |  | 16 479 |  | 4647 | 0.28 |  | 13 546 | 0.82 |  | 22.70 | 1378 |
| 22.5-<25 |  | 24 |  | 11 738 (19) |  | 44 882 |  | 14 345 | 0.32 |  | 33 521 | 0.75 |  | 62.30 | 1388 |
| 25-<27.5 |  | 26 |  | 15 964 (26) |  | 61 043 |  | 20 838 | 0.34 |  | 45 955 | 0.75 |  | 91.10 | 1492 |
| 27.5-<30 |  | 29 |  | 13 170 (22) |  | 49 812 |  | 20 220 | 0.41 |  | 45 824 | 0.92 |  | 84.00 | 1686 |
| 30-<32.5 |  | 31 |  | 7606 (12) |  | 28 842 |  | 11 806 | 0.41 |  | 31 437 | 1.09 |  | 57.30 | 1987 |
| 32.5-<35 |  | 34 |  | 3765 (6) |  | 14 277 |  | 7252 | 0.51 |  | 17 839 | 1.25 |  | 31.00 | 2171 |
| 35-<40 |  | 37 |  | 2670 (4) |  | 10 033 |  | 5836 | 0.58 |  | 17 016 | 1.70 |  | 26.30 | 2621 |
| 40-50 |  | 42 |  | 875 (1) |  | 3275 |  | 2637 | 0.81 |  | 7520 | 2.30 |  | 10.30 | 3145 |
| **Total** |  | - |  | **61 088 (100)** |  | **232 381** |  | **89 143** | **0.38** |  | **217 585** | **0.94** |  | **392.18** | **1688** |
| **65-79 years** |  |  |  |  |  |  |  |  |  |  |  |  |  |  |  |
| 15-<18.5 |  | 18 |  | 189 (1) |  | 661 |  | 949 | 1.44 |  | 4426 | 6.70 |  | 4.26 | 6445 |
| 18.5-<20 |  | 19 |  | 385 (1) |  | 1367 |  | 1991 | 1.46 |  | 5997 | 4.39 |  | 7.80 | 5706 |
| 20-<22.5 |  | 22 |  | 2665 (8) |  | 9907 |  | 6967 | 0.70 |  | 27 309 | 2.76 |  | 39.00 | 3937 |
| 22.5-<25 |  | 24 |  | 7209 (22) |  | 27 015 |  | 21 525 | 0.80 |  | 65 962 | 2.44 |  | 103.00 | 3813 |
| 25-<27.5 |  | 26 |  | 9147 (28) |  | 34 328 |  | 26 043 | 0.76 |  | 79 727 | 2.32 |  | 128.00 | 3729 |
| 27.5-<30 |  | 29 |  | 6917 (21) |  | 25 882 |  | 22 431 | 0.87 |  | 69 200 | 2.67 |  | 109.00 | 4211 |
| 30-<32.5 |  | 31 |  | 3589 (11) |  | 13 369 |  | 12 376 | 0.93 |  | 39 229 | 2.93 |  | 63.00 | 4712 |
| 32.5-<35 |  | 33 |  | 1737 (5) |  | 6367 |  | 5918 | 0.93 |  | 22 115 | 3.47 |  | 33.60 | 5277 |
| 35-<40 |  | 37 |  | 1058 (3) |  | 3856 |  | 4290 | 1.11 |  | 14 474 | 3.75 |  | 21.60 | 5602 |
| 40-50 |  | 42 |  | 286 (1) |  | 1031 |  | 1280 | 1.24 |  | 5336 | 5.18 |  | 6.55 | 6353 |
| **Total** |  | - |  | **33 182 (100)** |  | **123 782** |  | **103 770** | **0.84** |  | **333 775** | **2.70** |  | **515.81** | **4167** |
| **≥80 years** |  |  |  |  |  |  |  |  |  |  |  |  |  |  |  |
| 15-<18.5 |  | 17 |  | 200 (2) |  | 619 |  | 680 | 1.10 |  | 4878 | 7.88 |  | 4.55 | 7351 |
| 18.5-<20 |  | 19 |  | 331 (3) |  | 1095 |  | 1674 | 1.53 |  | 8185 | 7.47 |  | 8.12 | 7416 |
| 20-<22.5 |  | 22 |  | 1829 (16) |  | 6320 |  | 9538 | 1.51 |  | 41 037 | 6.49 |  | 45.10 | 7136 |
| 22.5-<25 |  | 24 |  | 3358 (30) |  | 11 947 |  | 14 497 | 1.21 |  | 70 531 | 5.90 |  | 79.90 | 6688 |
| 25-<27.5 |  | 26 |  | 2880 (25) |  | 10 289 |  | 14 447 | 1.40 |  | 62 171 | 6.04 |  | 71.30 | 6930 |
| 27.5-<30 |  | 28 |  | 1593 (14) |  | 5741 |  | 7397 | 1.29 |  | 35 350 | 6.16 |  | 42.20 | 7351 |
| 30-<32.5 |  | 31 |  | 740 (7) |  | 2589 |  | 4143 | 1.60 |  | 17 791 | 6.87 |  | 19.90 | 7686 |
| 32.5-<35 |  | 33 |  | 264 (2) |  | 912 |  | 1199 | 1.31 |  | 6133 | 6.72 |  | 6.74 | 7390 |
| 35-<40 |  | 36 |  | 125 (1) |  | 435 |  | 814 | 1.87 |  | 3304 | 7.60 |  | 3.48 | 8000 |
| 40-50 |  | 44 |  | 27 (<0) |  | 91 |  | 70 | 0.77 |  | 438 | 4.81 |  | 0.56 | 6154 |
| **Total** |  | - |  | **11 347 (100)** |  | **40 036** |  | **54 459** | **1.36** |  | **249 818** | **6.24** |  | **281.85** | **7040** |
|  |  |  |  |  |  |  |  |  |  |  |  |  |  |  |  |
| **FEMALES** |  |  |  |  |  |  |  |  |  |  |  |  |  |  |  |
| 15-<18.5 |  | 18 |  | 1093 (1) |  | 4168 |  | 1168 | 0.28 |  | 3568 | 0.86 |  | 5.51 | 1322 |
| 18.5-<20 |  | 19 |  | 3114 (4) |  | 11 732 |  | 3581 | 0.31 |  | 8342 | 0.71 |  | 12.70 | 1083 |
| 20-<22.5 |  | 21 |  | 12 779 (16) |  | 48 560 |  | 13 938 | 0.29 |  | 28 902 | 0.60 |  | 52.20 | 1075 |
| 22.5-<25 |  | 24 |  | 17 511 (22) |  | 66 627 |  | 19 012 | 0.29 |  | 41 024 | 0.62 |  | 74.20 | 1114 |
| 25-<27.5 |  | 26 |  | 14 514 (19) |  | 55 118 |  | 17 861 | 0.32 |  | 39 013 | 0.71 |  | 68.60 | 1245 |
| 27.5-<30 |  | 29 |  | 10 385 (13) |  | 39 367 |  | 13 381 | 0.34 |  | 30 546 | 0.78 |  | 56.70 | 1440 |
| 30-<32.5 |  | 31 |  | 7491 (10) |  | 28 331 |  | 10 746 | 0.38 |  | 24 849 | 0.88 |  | 44.50 | 1571 |
| 32.5-<35 |  | 34 |  | 4599 (6) |  | 17 350 |  | 6965 | 0.40 |  | 17 734 | 1.02 |  | 31.30 | 1804 |
| 35-<40 |  | 37 |  | 4667 (6) |  | 17 671 |  | 7751 | 0.44 |  | 21 801 | 1.23 |  | 37.60 | 2128 |
| 40-50 |  | 43 |  | 2252 (3) |  | 8445 |  | 4555 | 0.54 |  | 14 535 | 1.72 |  | 22.10 | 2617 |
| **Total** |  | - |  | **78 405 (100)** |  | **297 368** |  | **98 958** | **0.33** |  | **230 314** | **0.77** |  | **405.41** | **1363** |
| **65-79 years** |  |  |  |  |  |  |  |  |  |  |  |  |  |  |  |
| 15-<18.5 |  | 18 |  | 553 (2) |  | 2029 |  | 1758 | 0.87 |  | 7503 | 3.70 |  | 7.86 | 3874 |
| 18.5-<20 |  | 19 |  | 1027 (3) |  | 3865 |  | 2250 | 0.58 |  | 8594 | 2.22 |  | 11.80 | 3053 |
| 20-<22.5 |  | 21 |  | 4052 (14) |  | 15 210 |  | 10 175 | 0.67 |  | 32 092 | 2.11 |  | 45.00 | 2959 |
| 22.5-<25 |  | 24 |  | 6625 (22) |  | 25 075 |  | 16 049 | 0.64 |  | 47 264 | 1.88 |  | 69.50 | 2772 |
| 25-<27.5 |  | 26 |  | 6167 (21) |  | 23 347 |  | 14 062 | 0.60 |  | 46 566 | 1.99 |  | 69.50 | 2977 |
| 27.5-<30 |  | 29 |  | 4648 (16) |  | 17 523 |  | 12 258 | 0.70 |  | 36 369 | 2.08 |  | 53.90 | 3076 |
| 30-<32.5 |  | 31 |  | 3064 (10) |  | 11 496 |  | 9627 | 0.84 |  | 28 533 | 2.48 |  | 40.80 | 3549 |
| 32.5-<35 |  | 34 |  | 1782 (6) |  | 6631 |  | 5668 | 0.85 |  | 18 133 | 2.73 |  | 25.60 | 3861 |
| 35-<40 |  | 37 |  | 1480 (5) |  | 5490 |  | 4665 | 0.85 |  | 18 188 | 3.31 |  | 24.30 | 4426 |
| 40-50 |  | 42 |  | 525 (2) |  | 1901 |  | 2189 | 1.15 |  | 7631 | 4.01 |  | 9.20 | 4840 |
| **Total** |  | - |  | **29 923 (100)** |  | **112 568** |  | **78 701** | **0.70** |  | **250 873** | **2.23** |  | **357.46** | **3176** |
| **≥80 years** |  |  |  |  |  |  |  |  |  |  |  |  |  |  |  |
| 15-<18.5 |  | 18 |  | 482 (5) |  | 1553 |  | 1857 | 1.20 |  | 11 718 | 7.55 |  | 10.30 | 6632 |
| 18.5-<20 |  | 19 |  | 739 (7) |  | 2495 |  | 2209 | 0.89 |  | 15 734 | 6.31 |  | 14.60 | 5852 |
| 20-<22.5 |  | 21 |  | 1902 (18) |  | 6590 |  | 6627 | 1.01 |  | 37 348 | 5.67 |  | 37.40 | 5675 |
| 22.5-<25 |  | 24 |  | 2547 (25) |  | 8927 |  | 8163 | 0.91 |  | 48 086 | 5.39 |  | 48.20 | 5399 |
| 25-<27.5 |  | 26 |  | 2016 (20) |  | 7130 |  | 6230 | 0.87 |  | 35 303 | 4.95 |  | 37.20 | 5217 |
| 27.5-<30 |  | 29 |  | 1237 (12) |  | 4366 |  | 4546 | 1.04 |  | 25 444 | 5.83 |  | 25.20 | 5772 |
| 30-<32.5 |  | 31 |  | 716 (7) |  | 2509 |  | 2103 | 0.84 |  | 14 107 | 5.62 |  | 13.70 | 5460 |
| 32.5-<35 |  | 34 |  | 349 (3) |  | 1250 |  | 1127 | 0.90 |  | 6728 | 5.38 |  | 7.05 | 5640 |
| 35-<40 |  | 37 |  | 245 (2) |  | 825 |  | 741 | 0.90 |  | 4720 | 5.72 |  | 4.93 | 5976 |
| 40-50 |  | 42 |  | 76 (1) |  | 271 |  | 712 | 2.63 |  | 1970 | 7.27 |  | 1.72 | 6347 |
| **Total** |  | - |  | **10 309 (100)** |  | **35 915** |  | **34 315** | **0.96** |  | **201 158** | **5.60** |  | **200.31** | **5577** |

Abbreviations: BMI=body mass index; n=number of participants; py=person year.

Notes. 1. Costs are derived from DRG-specific cost estimates in the 2009-10 National Hospital Cost Data Collection Public Sector Estimated Cost Weights Report (NHCDC).[[29](#_ENREF_29)]
