# Supplementary figures and images for: The Relationship between Body Mass Index and Hospitalisation Rates, Days in Hospital and Costs: Findings from a Large Prospective Linked Data Study
Source: PLoS One. 2015 Mar 4;10(3):e0118599. doi: 10.1371/journal.pone.0118599 (PMC4349828; doi:10.1371/journal.pone.0118599)

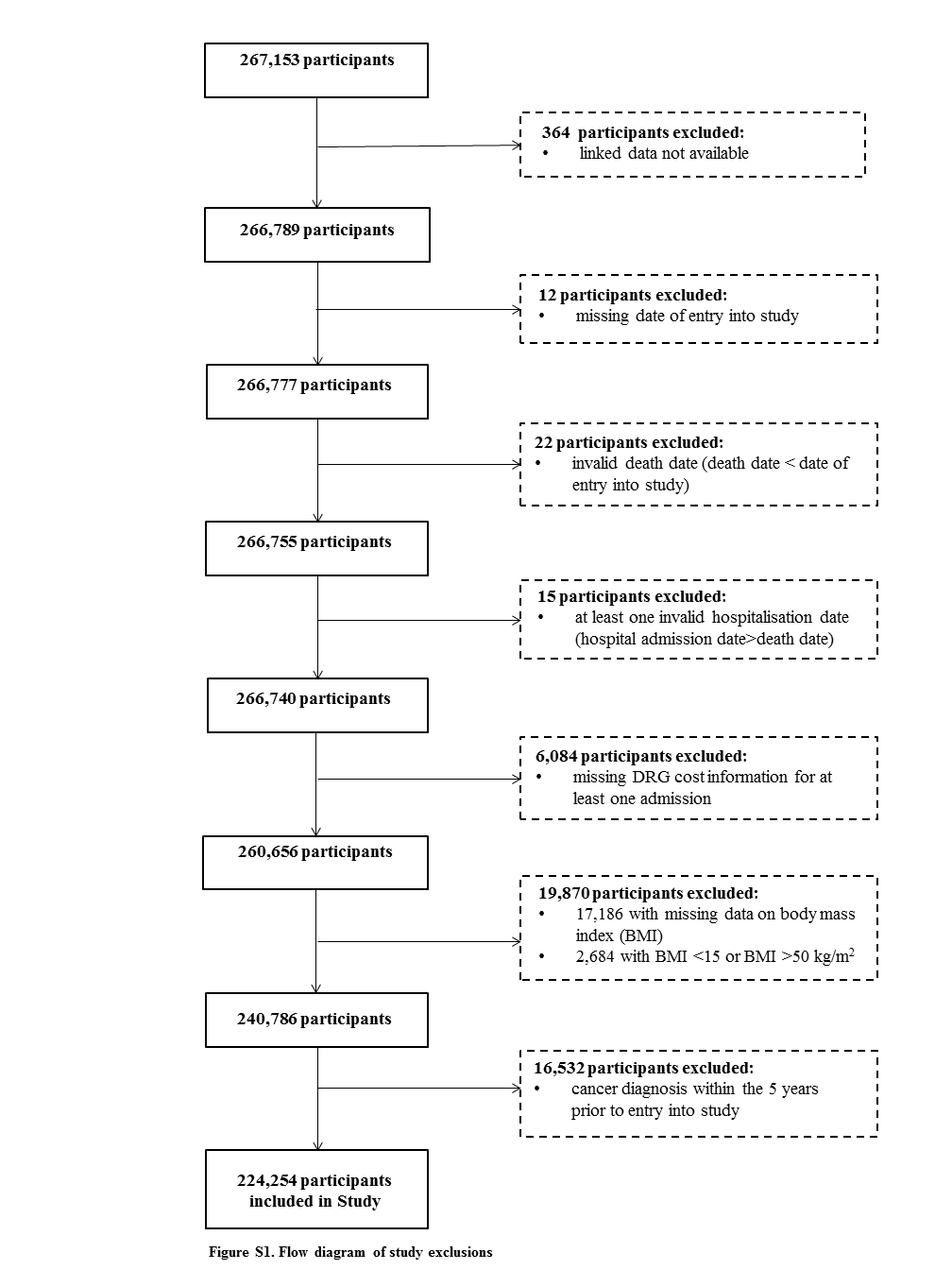

Supplement: S1 Fig — (TIF) [file pone.0118599.s003.tif]
